# Barriers to Infectious Disease Prevention among Women^1^

**DOI:** 10.3201/eid1011.040622_07

**Published:** 2004-11

**Authors:** JoAnn Thierry, Jeanne Marrazzo, Madeleine LaMarre

**Affiliations:** * Centers for Disease Control and Prevention, Atlanta, Georgia, USA;; † University of Washington, Seattle, Washington, USA;; ‡ Georgia Department of Corrections, Atlanta, Georgia, USA

**Keywords:** infectious disease, prevention, woman, conference summary

Barriers to infectious disease prevention for women can include healthcare access, physical inaccessibility, cultural and linguistic barriers, and prejudice or ignorance regarding the women's particular needs. These barriers are particularly evident for women with disabilities, women who have sex with women, and women who are incarcerated.

## Lesbians and Infectious Diseases

The term "lesbian" refers to a woman who engages in sex with another woman. In the United States, estimates of lifetime same-sex behavior among women are 8%–20%, and 1.4%–4.3% of all women may be sexually active with other women. An estimated 2.3 million women describe themselves as lesbian. In its 1999 report, Lesbian Health: Current Assessment and Directions for the Future, the Institute of Medicine emphasized that more data on sexually transmitted infections (STIs), Pap smear screening, and risk for cervical cancer in lesbians were needed (*1*).

The risk for STI transmission between women depends on the specific STI and on the sex practices involved. Bacterial vaginosis occurs in 24% to 51% of lesbians, frequently in both members of monogamous couples; sexual transmission has been postulated (*2*). Transmission of common STIs, especially human papillomavirus (HPV) and herpes simplex virus (HSV), and syphilis requires only skin-to-skin or mucosal contact. Most lesbians (53%–99%) have had sex with men, and many (21%–30%) continue to do so; they may acquire viral STIs from men and subsequently transmit them to female partners.

Routine Pap smear screening is probably performed less frequently among many lesbians than national guidelines suggest. Despite high levels of education and income, women with no prior sex with men were less likely to have ever received a pelvic examination, received their first Pap smear at an older age, and had less frequent Pap smears than women who had prior sex with men.

Potential barriers to preventive care by lesbians include lack of knowledge, insensitivity among providers, inability to pay (lack of insurance or lower earnings in households without at least one male), and perception of low risk for STI and cervical cancer by lesbians and providers.

Available data strongly suggest that HPV infection, and probably other STIs, are sexually transmitted between women. Thus, recommendations for Pap smear screening among lesbians should not differ from those for heterosexual women, a point that should be clearly communicated in guidelines and relevant training.

## Incarcerated Women and Infectious Diseases

The United States has the one of the highest incarceration rate in the world. This high rate may be directly related to the widespread prevalence of illicit drugs, which disproportionately affect impoverished populations. Disease is introduced into the prison population through imprisoned persons who have diseases and did not have adequate health care before incarceration.

Women are mainly incarcerated for drug-related offenses (72% of all offenses for women in federal prison in 1998). In a 2002 Georgia study of new inmates, 23% of all women were previously or acutely infected with hepatitis B virus; 19% of men were infected. From 1998 to 1999, the infection rate among women entering Georgia's correctional system was >11%. Chlamydia and gonorrhea are more prevalent among juvenile populations (15–19 years of age); HIV and syphilis are found more often in those >20 years.

Eighty-eight percent of state and federal correctional systems in the United States in 1997 reported syphilis screening. In 1997, only 16 of 51 state prisons reported that they screened all inmates for HIV; 25 reported that they screen only high-risk groups, symptomatic inmates, and inmates who request screening. Incarcerated women face challenges in receiving adequate health care because treatment and prevention of diseases is often not a top prison priority.
